# Spatial trends of breast and prostate cancers in the United States between 2000 and 2005

**DOI:** 10.1186/1476-072X-8-53

**Published:** 2009-09-29

**Authors:** Rakesh Mandal, Sophie St-Hilaire, John G Kie, DeWayne Derryberry

**Affiliations:** 1 Department of Health & Nutrition Sciences, Idaho State University, 921 South 8th Avenue, Stop 8109, Pocatello, ID 83209-8109, USA; 2 Department of Biological Sciences, Idaho State University, 921 South 8th Avenue, Stop 8007, Pocatello, ID 83209-8007, USA; 3 Department of Mathematics, Idaho State University, 921 South 8th Avenue, Stop 8085, Pocatello, ID 83209-8085 USA

## Abstract

**Background:**

Breast cancer in females and prostate cancer in males are two of the most common cancers in the United States, and the literature suggests that they share similar features. However, it is unknown whether the occurrence of these two cancers at the county level in the United States is correlated. We analyzed Caucasian age-adjusted county level average annual incidence rates for breast and prostate cancers from the National Cancer Institute and State Cancer Registries to determine whether there was a spatial correlation between the two conditions and whether the two cancers had similar spatial patterns.

**Results:**

There was a significant correlation between breast and prostate cancers by county (r = 0.332, p < 0.001). This relationship was more pronounced when we performed a geographically-weighted regression (GWR) analysis (r = 0.552) adjusting for county unemployment rates. There was variation in the parameter estimates derived with the GWR; however, the majority of the estimates indicted a positive association. The strongest relationship between breast and prostate cancer was in the eastern parts of the Midwest and South, and the Southeastern U.S. We also observed a north-south pattern for both cancers with our cluster analyses. Clusters of counties with high cancer incidence rates were more frequently found in the North and clusters of counties with low incidence rates were predominantly in the South.

**Conclusion:**

Our analyses suggest breast and prostate cancers cluster spatially. This finding corroborates other studies that have found these two cancers share similar risk factors. The north-south distribution observed for both cancers warrants further research to determine what is driving this spatial pattern.

## Background

**Figure 1:**
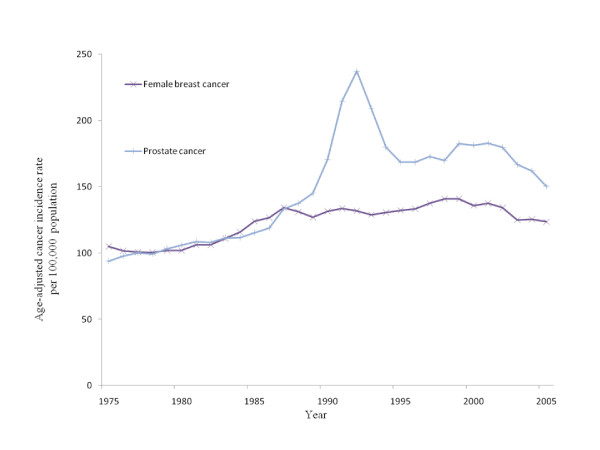
**U.S. average age-adjusted incidence rates for breast and prostate cancer from 1975 to 2005**. Data were obtained from the National Cancer Institute-Surveillance, Epidemiology and End Results.

Breast cancer in females and prostate cancer in males are two of the most common cancers in the United States. Besides a sudden spike in the rate of prostate cancer between 1989 and 1992, attributed to PSA screening [1] the incidence of both cancers over time has followed a similar pattern (Figure 1). The overall age-adjusted incidence rate of breast and prostate cancer in 2004 in the U.S. was 117.7 and 145.3 per 100,000 people, respectively [2]. Despite the similar national incidence figures for these cancers there is considerable variation in the rates of both cancers at the county level. For example, the highest and lowest incidence rates for breast cancer in 2004 were 345.5 and 29 cases per 100,000, respectively. For prostate cancer the range was between 346.2 and 44.2 cases per 100,000 [3].

An extensive review by López-otín and Diamandis compared breast and prostate cancers and highlighted several similar features and characteristics [4]. One of the most obvious similarities between breast and prostate cancers is their hormonal regulation. At least some breast and prostate cancer cell types appear to have receptors for a number of the same steroid hormones (e.g. estrogens, progesterone, and androgens) and growth hormones, such as androgen-induced growth factor and keratinocyte growth factor. The negative impact of high levels of endogenous sex steroids, and the benefit of low circulating sex steroids for both breast and prostate cancers is well documented in the literature [5,6], and suggests that exposure to exogenous hormones (i.e. hormone therapy, contraceptives, dietary fats, and environmental endocrine disruptors) may also have a negative impact on the onset and progression of these diseases. In fact, anti-estrogens and anti-androgens are sometimes effective treatments for breast and prostate cancers, respectively [7].

The patho-physiological mechanisms by which breast and prostate tumors develop is not well understood, but evidence suggests several independent pathways may exist, involving different receptors and complex cascades of events that ultimately culminate in abnormal cell proliferation. Most often tumors of the breast and prostate involve epithelial cell types and express similar biochemical markers, which suggests analogous patho-physiologies [4]. At least one of these common biomarkers-prostate specific antigen-has been detected in breast and prostate tumors, and in no other tumors [8].

Some of the main gene alterations associated with breast cancer (e.g. BRCA1 and BRCA2) have also been found in some individuals with prostate cancer [9], and the most commonly identified gene alteration in prostate cancer patients (e.g. alterations in the AR gene) has been detected in breast cancer patients [10]. The similarity in the genetic component of these two cancers suggests they share similar patho-physiological mechanisms. Another link between these two cancers is the epidemiological studies, which suggest individuals from families with a high incidence of breast cancer are more likely to develop prostate cancer and vice versa [11]. Interestingly, genetics accounts for about 5% of both breast and prostate cancer cases [12].

Epidemiological studies have also identified similar protective factors for both breast and prostate cancers. In the last 17 years vitamin D has received a great deal of attention as an important compound for both breast and prostate cancer prevention [13-16]. It is suggested that the active form of vitamin D, 1,25(OH)_2_D regulates transcription in cells with vitamin D receptors including breast and prostate cells [17].

These two types of cancers share many similarities, but their spatial distributions have not been compared. If they are homologous cancers they should occur in similar areas at similar rates. The objective of our study was to determine whether these two cancers are spatially correlated.

## Results

### Breast cancer clusters

**Figure 2:**
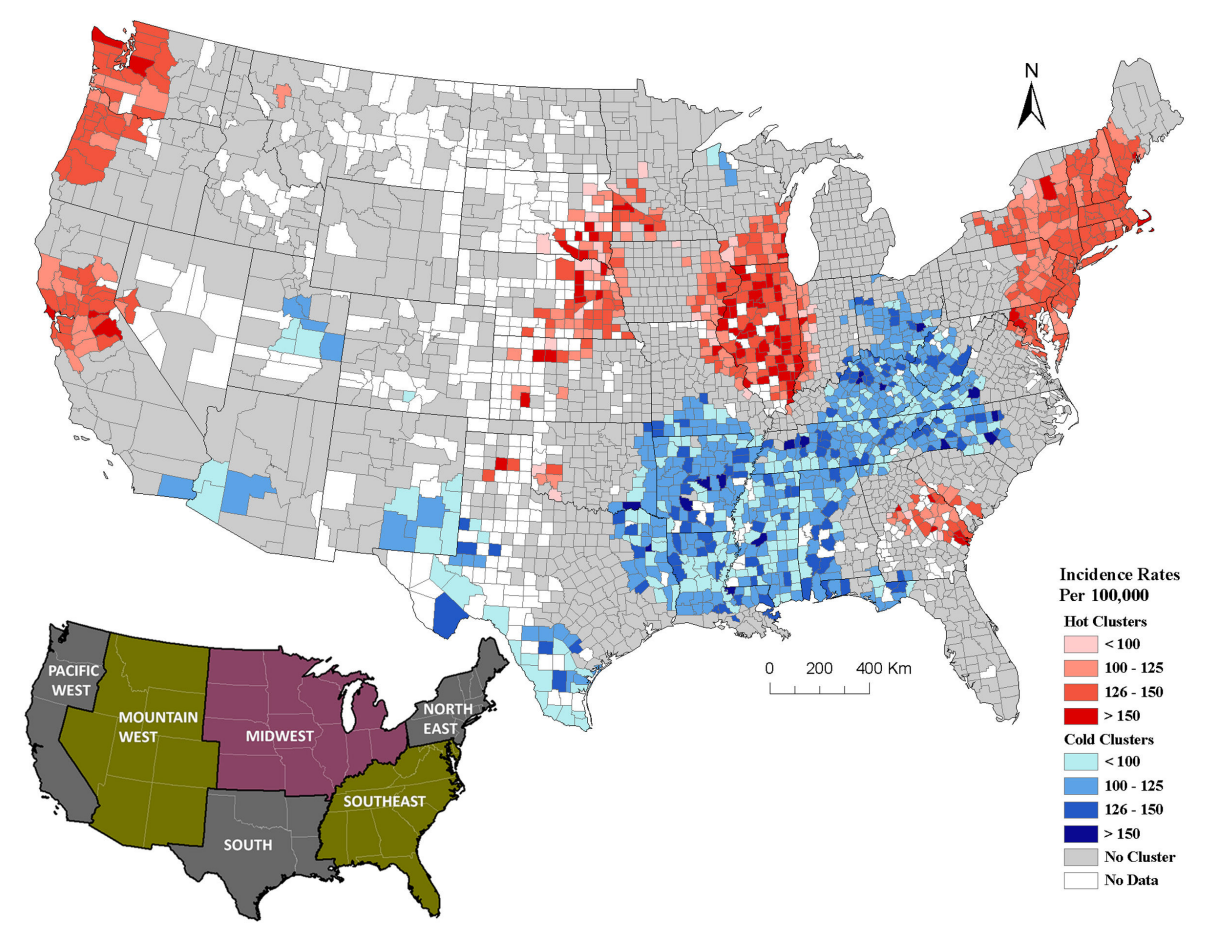
**Geographical clusters of U.S. counties with significant high or low breast cancer incidence rates among Caucasians (including Hispanics) analyzed at a 200 km distance band**. The graduated red and blue colors show high (hot) and low (cold) clusters respectively, for age-adjusted average annual incidence rates (2000/2001-2004/2005) of breast cancer. The counties with no color either have no data or counts less than 3-5. Graduated colors were assigned to the hot and cold clusters based on the incidence rate of individual counties. In total there were 2,692 counties used in the breast cancer cluster analysis. The inserted regional U.S. map depicts the regions of the U.S. used to describe the cluster patterns. Data source: National Cancer Institute-State Cancer Profiles and State Cancer Registries.

The Getis-Ord Gi* analysis suggests there were statistically signifcant clusters of counties with high incidence rates of breast cancer ("hot" clusters) in the Northeast, Midwest and northern and mid Pacific West regions (Figure 2). A medium size hot cluster occurred in the Southeast region, and a small cluster occurred in the northern part of the South region (Figure 2). The latter may have been an extension of the hot cluster in the Midwest had there not been missing data in that region (Figure 2).

"Cold" clusters (or areas where the incidence of breast cancer was relatively low) occurred predominantly in the South (Figure 2). There was only one small cold cluster in the northern Midwest (Figure 2).

### Prostate cancer clusters

**Figure 3:**
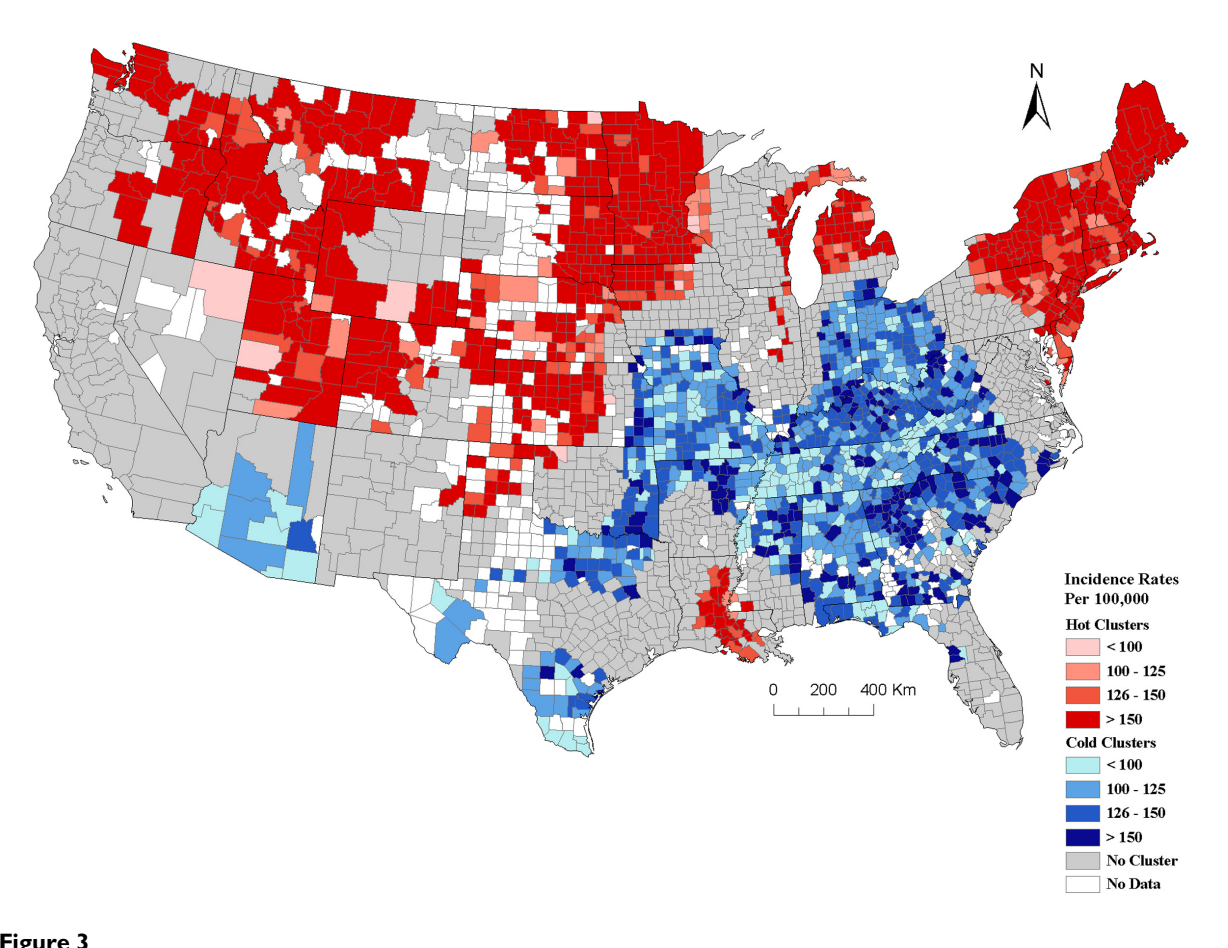
**Geographical clusters of U.S. counties with significant high or low prostate cancer incidence rates among Caucasians (including Hispanics) analyzed at a 200 km distance band**. The graduated red and blue colors show high (hot) and low (cold) clusters respectively, for age-adjusted average annual incidence rates (2000/2001-2004/2005) of prostate cancer. The counties with no color are those with either no data or counts less than 3-5. Graduated colors were assigned to the hot and cold clusters based on the incidence rate for counties. In total there were 2,777 counties used in the prostate cancer cluster analysis. Data source: National Cancer Institute-State Cancer Profiles and State Cancer Registries.

Overall, the incidence rate of prostate cancer also had a north-south distribution with the North having a high incidence of prostate cancer and the South having counties with a lower incidence rate, with one exception in Louisiana and Mississippi (Figure 3). There were large clusters of counties with a high incidence rate of prostate cancer in the Northeast, northern and western parts of the Midwest, northern part of the Mountain West regions, and the eastern part of the South region (Figure 3).

There was a large area where counties had a lower incidence of prostate cancer than expected. This area spanned the southern part of the Midwest and northern part of the South and Southeast regions (Figure 3). There were small to medium-sized cold clusters that also occurred in the southern parts of the Mountain West and South regions.

For the most part there was a north-south distribution to both types of cancers; however, the hot and cold clusters for these cancers did not always overlap. Shared geographic clusters with high incidence rates of breast and prostate cancers occurred in the Northeast and Midwest (Figures 2 and 3). Common areas of cold clusters for both cancers were found in the South, parts of the Southeast region, and southern parts of the Mountain West region (Figures 2 and 3). The north-south distribution for both cancers was observed regardless of the band distance used in the Getis-Ord Gi* cluster analysis.

### Correlations

The least squares correlation coefficient for the dataset on Caucasians with breast and prostate cancer was 0.332. The correlation coefficient for breast and prostate cancers for the smaller subset of data for all races was 0.336 (Table 1). The latter coefficient was higher than the coefficients with the other cancers and breast and prostate cancers (r was always less than 0.149; Table 1).

**Table 1:** Correlation coefficients for regression analyses between different types of cancers.

**Cancer types**		**Correlation (r)**	**Number of counties**	**Race/Ethnicity**	**P Value**
Breast	Prostate	0.332	2651^a^	Caucasians & Hispanics	< 0.001

Breast	Prostate	0.336	2205 ^b^	All races	< 0.001

Breast	Colon/Rectum	0.139	1804 ^b^	All races	< 0.001

Breast	Lung/Bronchus	0.149	1845 ^b^	All races	< 0.001

Breast	Ovary	0.106	801 ^b^	All races	0.003

Prostate	Lung/Bronchus	-0.018	2070 ^b^	All races	0.41

Prostate	Colon/Rectum	0.124	1859 ^b^	All races	< 0.001

Ordinary least square regressions analyses were conducted on age-adjusted (to the 2000 U.S. standard population) county level average incidence rates (cases per 100,000 population per year) for different types of cancers. Data used in these analyses were extracted from the National Cancer Institute (NCI) and State Cancer Registries.

^a^Data from counties in all states were included for the period between 2000 (or 2001) and 2004 (or 2005).

^b ^Data from counties in all states except Illinois, Maryland, Minnesota, Mississippi, North Dakota, Tennessee and Virginia were included for the period between 2000 (or 2001) and 2004.

The correlation coefficient from the unemployment-adjusted geographically-weighted regression (GWR) analysis for breast and prostate cancer incidence rates for Caucasians was 0.552, which suggests a stronger correlation between the two cancers when information from the surrounding counties was taken into account. There were only 26 out of 2651 (1.0%) counties that had standardized residuals greater than or less than 3 standard deviations from the mean. This was only slightly above what is expected from normal variation suggesting the regression model fit the data well. Further, the counties with these more extreme residual values appeared to be dispersed at random throughout the U.S.

**Figure 4:**
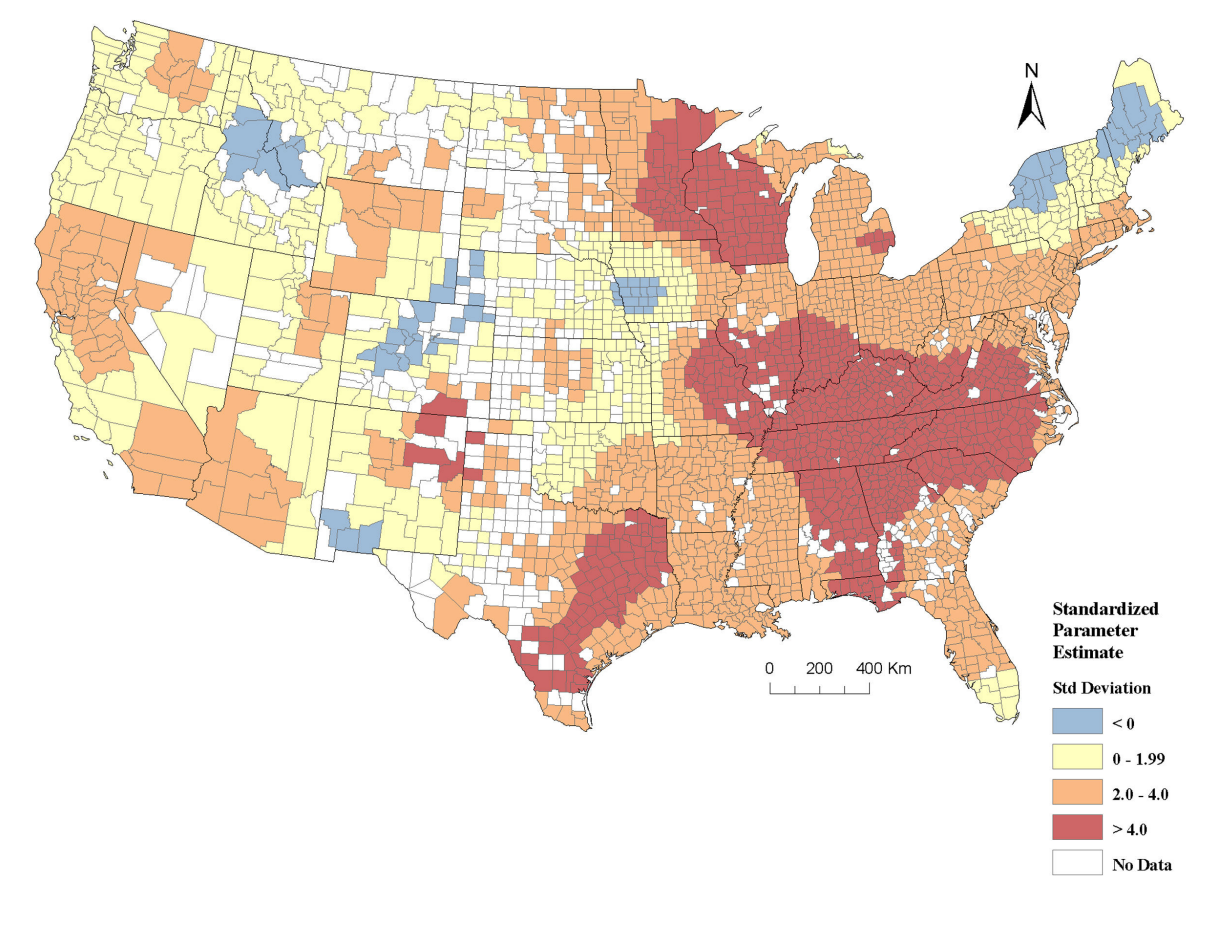
**County level standardized parameter estimates from a geographically-weighted regression, analyzed at a 200 km distance band comparing age-adjusted average annual incidence rates (2000/2001-2004/2005) of breast and prostate cancers adjusting for the county unemployment rate**. In total there were 2651 counties included in the analysis. The counties with no color are those with either no data or counts less than 3-5. Counties depicted in orange and red indicate statistically significant positive parameter estimates (i.e. positive relationship between the two cancers). Data sources: National Cancer Institute-State Cancer Profiles and State Cancer Registries and USDA Economic Research Service. (Std Standard).

The standardized county-level parameter estimates from the GWR were mostly positive (97.6%), which indicates a positive association between breast and prostate for most counties. Further, the negative standardized parameters were not statistically significant (i.e. not less than -2 standard deviations from the mean), and 76.2% of the positive values were significant (i.e. greater than 2 standard deviations from the mean). The pattern obtained by mapping the standardized parameter estimate suggests the strongest relationship between breast and prostate cancer was in the eastern parts the Midwest and South, and in the Southeastern U.S. (Figure 4).

## Discussion

We determined, using county-level data from the NCI, that the annual age-adjusted incidence rates of breast and prostate cancer in the U.S. between 2000/2001 and 2004/2005 were correlated at the county level (Table 1 and Figure 4). In general, counties with a high incidence of breast cancer also had a high incidence of prostate cancer, and vice versa. The correlation coefficient between these two cancers was greater than the correlation coefficient between these cancers and other cancers that are not hormonally regulated (Table 1), suggesting that risk factors for both breast and prostate cancers either cluster together spatially or the two cancers share common risk factors.

The correlation between these cancers increased from 0.332 to 0.552 when we used a geographically-weighted regression model, which accounted for data within a 200 km radius. This sudden increase in the correlation coefficient suggests similar risk factors for these cancers at a geographical area greater than the county level. These results also suggest our county level correlation is unlikely to be due to the county's cancer detection and reporting system.

The parameter estimates calculated for each county in our geographically-weighted regression model indicated over 76% of the counties had a significant positive association between breast and prostate cancer. This relationship varied across the U.S. and was strongest in the eastern area of the Midwest and adjacent areas of the Southeast and Southern U.S (Figure 4). The areas, where the standardized parameter estimates were the highest, were often where the hot and cold clusters for breast and prostate cancers overlapped (Figures 2, 3, and 4). There were only a few areas where the parameter estimates suggested a negative correlation between breast and prostate cancer, and these values were not statistically significant (i.e. blue areas in Figure 4). For the most part, our data suggested the rates for both of these cancers were positively correlated. This study identifies counties, as well as larger geographic areas within the U.S. where this correlation is strongest and weakest, which is useful for further research into potential factors driving the incidence of these cancers.

Both cancers also had a distinct north-south distribution (Figures 2 and 3), with the exception of the area known as "cancer alley" in the states of Louisiana and Mississippi [18]. In general, areas with higher than expected incidence of cancer (hot clusters) were located in the northern states and areas with lower than expected incidence of cancer (cold clusters) were in the southern states. This trend has also been reported by Schwartz and Hanchette [19] for prostate cancer mortality rates in the U.S. A U.S.-wide spatial analysis has not been reported for breast cancer; however, there have been reports of higher occurrence of breast cancer mortality in the northeastern U.S. than the southeastern part of the country [20,21].

There are several possible explanations for the north-south pattern of breast and prostate cancers. One explanation proposed by several researchers is the low exposure to ultraviolet radiation (UV) in the northern states, especially during the winter months [19,22], which is believed to result in lower vitamin D levels [19]. There are several independent researchers who have experimentally documented the beneficial effects of vitamin D on differentiation and proliferation for cell types with vitamin D receptors such as prostate and breast cells [23,24]. There are also several epidemiological studies that have examined UV exposure as a modifying factor for breast and prostate cancers and found a protective effect [19,24].

Another risk factor that may contribute to the clustering of cancer in the North may be low temperature, which almost always confounds UV exposure. That is, areas with a high UV index generally have high temperature and those with a low UV index have lower temperature. Temperature has a significant effect on ecological processes. Experiments have demonstrated that the biodegradation of certain organic compounds, including endocrine disruptors and chelation of heavy metals, is temperature-dependant and slower at colder temperatures [25,26]. It is also documented that semi-volatile organic chemicals (i.e. PCBs) precipitate out of the atmosphere more efficiently at cold temperatures and during snow events [27-32]. There may, therefore, be an interaction between precipitation, temperature, and atmospheric pollution, and exposure to endocrine disruptors, which have been associated with an increase in risk of both breast and prostate cancer [6,33,34], may be greater at higher altitudes and latitudes. This phenomenon would occur on a global scale and may explain the higher incidence of cancers at higher latitudes that have been reported in numerous countries [24].

There are also other differences in the distribution of risk and protective factors across the U.S. that may partially explain the north-south distribution of cancer observed in this study. For example, cultural differences that increase or decrease the risk of cancer (i.e. behavior and diets) may be unevenly distributed between the northern and southern U.S. It is also possible that the rate of other diseases, such as cardiovascular disease, is higher in the southern U.S. [35] thereby resulting in premature mortality and lower incidence of cancer in these areas. Because this study was an ecological study and data were obtained at the county level, we could not adjust for differences in individual risk factors. However, we were able to adjust for age and race by using Caucasians only and age-adjusted rates in our analyses. So it is unlikely that age and race played a significant role in the distribution pattern observed.

Ethnicity may have contributed to the distribution pattern as we could not obtain data on Caucasians that were not of Hispanic origin. Because individuals of Hispanic origin have lower risks of breast and prostate cancers [2], and the distribution of individuals that are of Hispanic origin is not even throughout the continental U.S., this factor may have contributed to the north-south distribution pattern. However, it is unlikely that this factor was the only reason for the north-south distribution because other researchers have noted a similar pattern in other countries [24].

Socioeconomic status is a known risk factor for many cancers and their outcome. To minimize the effect of this variable on our outcome of interest we used incidence data instead of mortality data. Although socioeconomic status is associated with the detection of cancer, it is most likely less dependent on the availability of adequate health care than mortality rates, which is strongly influenced by the treatment received by the patient. We also corrected for this variable in our GWR model by including the county's average annual unemployment rate between 2001 and 2004. Despite this, it is still possible that this parameter biased the findings of the correlation and influenced the cluster analyses. However, the fact that other types of cancers were not as strongly correlated with breast and prostate cancers at the county level (Table 1) suggests breast and prostate cancers are correlated (i.e. counties with high incidence rates of breast cancer also tend to have a high incidence rate of prostate cancer and vice versa) regardless of the effect of socioeconomic status.

One other possible bias in this study was the disparity in the size of the counties within the continental U.S. In general, the counties in the east were much smaller than those in the west, which may have affected our cluster analyses. Because the predominant pattern observed was north to south, and this pattern was consistent using different distances to measure clusters (data not shown), we felt that the east to west variation in the size of individual counties most likely did not affect our overall conclusions. Further, the north-south disease pattern observed in this study is consistent with other research that has found a relationship between latitude and breast and prostate cancers in other areas of the world [24].

There were a few inconsistencies in the spatial distribution and correlation between breast and prostate cancers. For example, there was a small cluster of counties in the south known as "cancer alley" that had a high incidence of prostate cancer, but did not have higher than expected breast cancer rates. Similarly, there were a few clusters of counties with a high incidence of breast cancer in the southeast that did not coincide with elevated prostate cancer (Figure 2 and 3). The variation in the parameter estimates from our GWR analysis also suggests the relationship between these cancers varies and, therefore they may not be completely homologous. If we had refined our case definition and only included specific types of breast or prostate cancers that are more likely to be analogous (i.e. similar cell types and responsive to specific types of steroid hormones), the distribution may have overlapped better. Further, the aggregation of data at the county level renders it impossible to analyze information at a smaller spatial scale. The reason we used county level data is because it was age-adjusted, averaged over several years, and readily available for the entire U.S.

There are multiple factors that may act synergistically on prostate and breast cell types, while others may act antagonistically on these tissues [4,6], which may account for some of the inconsistencies in the distribution of the two types of cancers. Risk factors for these cancers may also not be equally distributed within the male and female populations in a county. Despite the differences in the distribution of these cancers, the distinct north-south spatial pattern and the positive correlation between the cancers warrants further investigation to identify the factors driving these patterns. A model that includes variables such as socioeconomic status, incidence of other diseases, temperature, precipitation, pollution and UV indices, and controls for ethnicity would provide insight into the epidemiology of breast and prostate cancers. The findings of this study add to the growing evidence in the literature that prostate and breast cancers have similar risk factors and patho-physiological mechanisms.

## Methods

### Spatial cluster analyses for individual cancers

We extracted age-adjusted (to the 2000 U.S. standard population) annual incidence rates (cases per 100,000 population per year) for breast and prostate cancer between 2000 and 2004 or 2001 and 2005 from the National Cancer Institute (NCI) website [3], for each county in the United States for Caucasians and Caucasians of Hispanic origin. All data from the NCI website originate from individual State Cancer Registries. Analyses were only performed on data for Caucasians (of Hispanic and non-Hispanic origin combined) with the exception of data from counties in Illinois. The data for this state were only available for all races combined; therefore, we only included the data from counties where more than 95% of the population was Caucasian. We assumed the rates were representative of Caucasians in these cases. Rates for prostate and breast cancer were only for invasive cancers (not in situ). We excluded counties with average annual counts of less than 3-5 from the analysis because stable accurate age-adjusted rates were not available for these counties.

For six states, including Illinois (2001-2005), Maryland (2000), Minnesota (2000-2004), Mississippi (2003-2005), Tennessee (1999-2003) and Virginia (2000-2004), we obtained data from individual State Cancer Registry websites, as their data were not available through the NCI. The time block used to calculate the average annual age-adjusted incidence rate varied slightly by states (i.e. 2000-2004 or 2001-2005, and in one case 1999-2003).

We assessed the cancer data from the continental United States for spatial clustering using the Getis-Ord Gi* ArcGIS (v 9.3). All counties with missing data were removed for this analysis. We used the fixed distance band of 200 km in the Getis-Ord Gi* cluster analysis for breast and prostate cancers. This distance was selected based on the autocorrelation detected using a semivariance analysis [36] (data not shown) and the Getis-Ord Gi* analysis algorithm criterion of at least 1 county and preferably 8-10 counties for reliable results. All counties with significant Z scores (e.g. values ≥ 1.96 and ≤ -1.96) were identified. Negative values represented counties where there were 200 km radius clusters of lower than expected cancer incidence rates and high Z scores indicated counties where there were higher than expected cancer incidence rates within a 200 km radius. We also ran the Getis-Ord Gi* analysis using a distance band of 300 km and 400 km for comparison. We used the regions of the U.S. depicted in Figure 2 to describe the cluster patterns. All maps were generated in ArcGIS (v 9.3).

### Spatial correlation between breast and prostate cancer

Two methods were used to assess the spatial correlation between the incidence rates of breast and prostate cancers. First, we used an ordinary least square regression model to determine if there was a correlation between breast and prostate cancers at the county level. Second, we used a geographically-weighted regression analysis to determine if there was correlation between the incidence rates of these two cancers at the county level after adjusting for local, spatially-structured variation.

### Ordinary least square regression analyses (OLS)

We used the dataset described above to assess whether counties with a high incidence of breast cancer also had a high incidence of prostate cancer and vice versa. The regression analyses were conducted in Minitab (v 15.1). Assumptions of parametric tests were tested using regression diagnostics.

For comparison, correlations between these two cancers and lung and bronchial, colon and rectal, ovarian, and testicular cancers were also calculated. The data on these cancers were extracted in a similar manner with the exception that all races were included in the calculations and the seven states that did not provide data to the NCI were excluded. To ensure an appropriate comparison we also extracted breast and prostate cancer incidence rates from these same states for all races. Regression analyses were conducted as described above.

### Geographically-weighted regression analyses (GWR)

We tested for and found spatial autocorrelation in the incidence of breast and prostate cancers among counties using a semivariance analysis [36], and concluded that incidence rates in nearby counties were more likely to be similar than among counties separated by greater distances. Our Getis-Ord Gi* cluster analyses also supported this finding. To evaluate the correlation between breast and prostate cancers accounting for data in surrounding counties, we conducted a geographically-weighted regression analysis using ArcGIS (v 9.3) [37] adjusting for county average annual unemployment rates between 2001 and 2004. The unemployment rates were obtained from the United States Department of Agriculture Economic Research Service [38]. Specifically, a fixed kernel type function with a 200 km bandwidth parameter was used to calculate the GWR regression coefficients. Standardized residuals greater than 3 standard deviations above and below the mean were identified. The parameter estimates, derived for each county were divided by their standard errors, creating standardized t statistics. These values were mapped in ArcGIS (v 9.3). The analysis was done using the same dataset used for the spatial clustering analysis on Caucasians, which included individuals of Hispanic origin.

## Competing interests

The authors declare that they have no competing interests.

## Authors' contributions

RM extracted the data, conducted the spatial analyses and drafted the manuscript. SS provided the idea for the project, assisted with the data interpretation, and helped write the manuscript. JGK and DD assisted with the statistical analyses and interpretation. All authors participated in the review and final approval of the manuscript.
